# A Molecular–Protein Fusion Framework for Rapid Virtual Screening: Accelerating Lead Discovery for “Undruggable’’ Oncogenic Targets

**DOI:** 10.3390/ph19050753

**Published:** 2026-05-12

**Authors:** Chenxi Zhou, Yanni Zhu, Chenrui Yang, Yu Gao, Jianyang Lu, Dengming Ming

**Affiliations:** 1 College of Biotechnology and Pharmaceutical Engineering, Nanjing Tech University, 30 South Puzhu Road, Jiangbei New District, Nanjing 211816, China; 202321166060@njtech.edu.cn (C.Z.);; 2 College of Computer and Information Engineering, School of Artificial Intelligence, Nanjing Tech University, Jiangbei New District, Nanjing 211816, China; 3 College of Mechanical and Power Engineering, Nanjing Tech University, Jiangbei New District, Nanjing 211816, China

**Keywords:** KRAS G12D, pancreatic ductal adenocarcinoma, deep learning, graph neural network (GNN), drug screening

## Abstract

**Background/Objectives**: KRAS G12D is one of the most frequent oncogenic mutations in pancreatic ductal adenocarcinoma (PDAC) and remains challenging to target because of its limited druggable binding pockets. This study aimed to develop a machine learning-based framework for rapid virtual screening of potential KRAS G12D inhibitors. **Methods**: A molecular–protein fusion prediction framework, MPFF-IS, was constructed by integrating the ESM2 protein language model with an MPNN-GNN molecular graph network to enable joint representation learning of protein and compound features. The model was trained using a KRAS G12D inhibitor dataset and applied to screen compounds from multiple chemical libraries. AutoDock Vina docking and 300 ns GROMACS molecular dynamics simulations were subsequently performed for structural validation. **Results**: MPFF-IS achieved favorable predictive performance on the test dataset and identified 2663 candidate compounds from more than 134,000 screened molecules. Several candidate ligands exhibited favorable binding affinity, stable proteinligand interactions, and enhanced structural stability compared with reference inhibitors, including MRTX1133 and BI-2852. Molecular dynamics analyses further supported the stability of the predicted complexes and the involvement of key binding residues within the KRAS G12D pocket. **Conclusions**: These findings demonstrate that MPFF-IS can efficiently identify potential KRAS G12D inhibitors and may provide a useful computational framework for precision drug discovery targeting difficult oncogenic proteins.

## 1. Introduction

Cancer remains one of the leading causes of death worldwide [1]. Among all cancers, pancreatic cancer has one of the lowest five-year survival rates, at only 11% [2]. Because pancreatic cancer is difficult to detect, patients are often diagnosed at an advanced stage [3]. In pancreatic ductal adenocarcinoma (PDAC), the most common driver mutations include the oncogene KRAS and the tumor suppressor genes CDKN2A, SMAD4, and TP53 [4,5], as well as a variety of other genes with lower mutation frequencies [6]. Among these, KRAS mutations are present in more than 95% of PDAC cases, making it a major driver of PDAC pathogenesis (86–91%) [7].

Ras proteins are a family of small GTPases that regulate cell proliferation, differentiation, and migration and play critical roles in cancers such as adenocarcinoma [7]. Upon activation of receptor tyrosine kinases (e.g., EGFR), KRAS transmits growth signals through the GTP/GDP cycle and activates signaling pathways such as RAF–MEK–ERK and PI3K–AKT1–mTOR [8,9,10,11,12]. KRAS mutations (e.g., G12D, G12V, and G12C) lead to constitutive activation of KRAS, which in turn promotes aberrant signal transduction and tumorigenesis. Among these, G12 mutations account for 89% [13,14,15], and G12D is the most common subtype in PDAC (45%) [7].

KRAS is difficult to target therapeutically because its nucleotide-binding site is not amenable to competitive inhibition and it lacks suitable allosteric binding pockets for inhibitors [11]. KRAS consists of a hypervariable region (HVR) and a catalytic domain, in which Switch I and Switch II regulate its activity [16]. The KRAS G12C mutation can create a hydrophobic pocket in Switch II, enabling binding by covalent inhibitors [17,18]. In contrast, G12D lacks a cysteine residue amenable to targeting, making therapeutic intervention more challenging [6].

For the G12D mutation, researchers developed MRTX1133, which selectively and reversibly binds to KRAS G12D, blocks RAF1 binding, and inhibits KRAS signaling, thereby significantly suppressing tumor growth in mouse models [19]. BI-2852 targets the Switch I and Switch II regions and reduces the levels of pERK and pAKT (IC50 = 450 nM) [20], while other inhibitors (such as TH-Z827 and TH-Z835) exert their effects by forming salt bridges with Asp12 [21].

Although research on KRAS G12D-targeted inhibitors has progressed rapidly, no related drug had been approved by the FDA for clinical use as of 2025. Meanwhile, new drug development is time-consuming and costly, whereas computer-aided drug design (CADD) methods can improve screening efficiency [11,22]. With advances in computational power, the application of artificial intelligence (AI) and machine learning (ML) to lead compound discovery has continued to expand, accelerating new drug development [16,23,24]. At present, most researchers employ traditional machine learning methods, such as RF, SVM, and KNN, to manually extract compound features and predict the binding strength between screened compounds and KRAS G12D, thereby determining whether a compound may serve as a potential inhibitor. Amar Ajmal et al. [20] developed four models using open-source Python v3.9, including k-nearest neighbors, support vector machines, naïve Bayes, and random forests, implemented with the Scikit-learn library. Ultimately, the random forest model achieved an accuracy of 98% on both the training and test sets. Its MCC values were 0.95 on the training set and 0.97 on the test set, making it the best-performing model. The authors also used the random forest model to predict potential KRAS G12D inhibitors and subsequently subjected them to molecular dynamics simulations.

Although these methods have identified many potential inhibitors with relatively high accuracy, docking-based predictions are prone to generating false positives [25]. In addition, traditional machine learning methods rely heavily on manually engineered features and often fail to capture complex protein–ligand interactions, which may limit their ability to generalize. Therefore, we designed the Molecular–Protein Fusion Framework for Inhibitor Screening model (MPFF-IS) to assess whether screened compounds inhibit the target, thereby reducing the likelihood of false positives.

In this framework, we employ an MPNN-GNN deep learning model as the backbone to learn compound representations, reducing reliance on labor-intensive manual feature engineering and enabling the extraction of more informative, previously unrecognized molecular features, thereby improving predictive accuracy. Meanwhile, both protein targets and compounds are incorporated as joint inputs, and a cross-coupled update mechanism is introduced to model their interactions. Compound features are iteratively refined at each update step, and protein and compound representations are subsequently fused for activity prediction. Compared with conventional single-input, compound-based models, this dual-input, interaction-aware design enables more effective characterization of protein–ligand relationships, leading to improved model accuracy and screening performance.

Accordingly, this study presents a graph convolution-based strategy for screening KRAS G12D-targeted inhibitors in pancreatic cancer. Compounds were first collected from the IBS, NPASS, ChEMBL, and ZINC databases using physicochemical filtering criteria and then screened with the MPFF-IS model to identify high-confidence candidates. These compounds were further evaluated through molecular docking at the binding sites of representative inhibitors MRTX1133 and BI-2852, followed by 300 ns molecular dynamics simulations of the top-ranked complexes to assess their stability. The results demonstrate that several newly identified compounds exhibit more stable binding than the reference inhibitors, suggesting their potential as KRAS G12D-targeted inhibitors. Overall, this study provides an effective computational framework for identifying candidate inhibitors and offers a practical strategy for drug discovery targeting challenging oncogenic proteins.

## 2. Results and Discussion

We constructed a predictive model based on the experimental dataset to screen potential KRAS G12D inhibitors. The model can identify compounds associated with KRAS G12D activity and capture molecular features that are difficult to recognize intuitively, providing a basis for subsequent screening. The selected candidates were further evaluated using molecular docking, in which compounds with unstable binding or unreasonable conformations were eliminated. Finally, representative hit compounds were subjected to molecular dynamics simulations to assess the stability and dynamic behavior of the compound–KRAS G12D complexes. The results support the reliability of the screened candidates and validate their potential inhibitory activity.

### 2.1. Model Results

#### 2.1.1. Model Training

During model construction, Bayesian optimization was employed for hyperparameter tuning. Based on prior training experience, the hyperparameter search space was defined as follows: the learning rate was set within the range [1 × 10^−4^, 1 × 10^3^] (linear scale), and the weight decay was set within [1 × 10^−4^, 1 × 10^−3^]. (linear scale). The batch size was selected from [64, 128], and the number of training epochs ranged from 100 to 300. Through iterative training and evaluation, the optimal hyperparameter combination was determined as: learning_rate = 4.8144 × 10^−4^, weight_decay = 5.9273 × 10^−3^, batch_size = 64, and epochs = 200.

**Figure 1:**
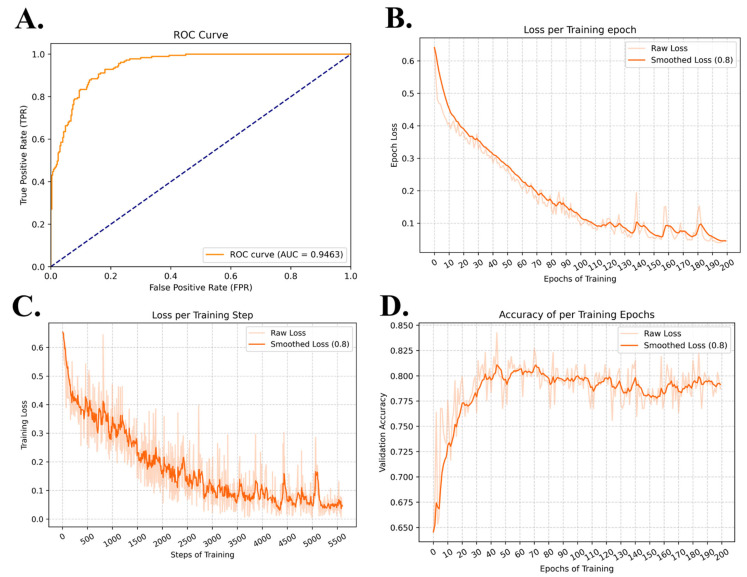
Training process and results. (**A**) ROC-AUC curve of the model on the general dataset test set; (**B**) Average loss curve per epoch on the general dataset; (**C**) Loss curve per training step on the general dataset; (**D**) Validation accuracy curve per epoch during training on the general dataset.

Based on these optimized hyperparameters, the model was retrained and validated, yielding the final trained model. Its performance on the test set is summarized in Table 1, while the training and validation loss and accuracy curves are presented in Figure 1.

The ROC curve (Figure 1A) shows a rapid increase in the true positive rate at low false positive rates, indicating strong discriminative ability of the model. The AUC of 0.9463 further demonstrates that the model achieves high classification performance and effectively distinguishes between positive and negative samples.

The loss curve per training epoch (Figure 1B) exhibits a steady downward trend, with the smoothed loss decreasing consistently as training progresses. This indicates that the model converges effectively during training. Minor fluctuations observed in the later stages are expected and suggest that the model has reached a stable optimization region rather than overfitting.

The loss per training step (Figure 1C) shows a similar decreasing trend, although with more pronounced fluctuations due to batch-level variability. The smoothed curve highlights a clear convergence pattern, indicating that the optimization process is stable and the model continues to learn meaningful representations throughout training.

The validation accuracy curve (Figure 1D) increases rapidly in the early epochs and gradually stabilizes around a high value. This trend suggests that the model quickly captures the underlying patterns in the data and maintains stable generalization performance. The absence of a significant decline in validation accuracy indicates that overfitting is effectively controlled.

The model outputs two types of predictions: (1) continuous probability values obtained after applying the Sigmoid function, and (2) binary classification labels, where samples with probabilities greater than 0.5 are classified as active. Analysis of the test set shows that samples with probability values closer to 1 tend to have higher prediction accuracy, while accuracy decreases as the probability declines. This suggests that the predicted probability can serve as an indicator of model confidence, and samples with values close to 1 are considered more reliable for subsequent analysis.

Table 1 presents the model’s performance evaluation results on the KRAS mutation dataset. The predictive performance of the model was evaluated using a scaffold-based data-splitting strategy, in which the dataset was divided into training, validation, and test sets in an approximate 8:1:1 ratio to avoid information leakage. The model was trained on the training set, hyperparameters were optimized on the validation set, and the final performance was assessed on the independent test set. For comparison, we reproduced and implemented traditional machine learning methods reported by Ajmal [26,27], Nadee [28], and others in KRAS G12D-related studies, and evaluated them on the same dataset. In addition, two representative deep learning drug–target interaction (DTI) models, DeepDTA [29] and GraphDTA [30], were included as baseline methods to comprehensively compare the predictive performance of different approaches on this task.

For quantitative evaluation, multiple metrics were employed, including accuracy, ROC-AUC, F1 score, MCC, recall, precision, and specificity. Accuracy reflects the overall classification performance and is defined as

$$Accuracy=\frac{TP+TN}{TP+TN+FP+FN}$$

ROC-AUC measures the model’s ability to distinguish between positive and negative samples across different thresholds, with values closer to 1.0 indicating stronger discriminative power. The F1 score balances precision and recall and is defined as

$$F1=\frac{2\times (Precision\times Recall)}{Precision+Recall}$$

MCC provides a comprehensive evaluation by considering all elements of the confusion matrix:

$$MCC=\frac{TP\times TN-FP\times FN}{\sqrt{\left(TP+FP)(TP+FN)(TN+FP)(TN+FN\right)}}$$

Recall measures the proportion of true positives correctly identified:

$$Recall=\frac{TP}{TP+FN}$$

Precision indicates the reliability of positive predictions:

$$Precision=\frac{TP}{TP+FP}$$

Specificity measures the proportion of true negatives correctly identified:

$$Specificity=\frac{TN}{TN+FP}$$

Here, TP (true positives) and TN (true negatives) denote correctly classified positive and negative samples, respectively, while FP (false positives) and FN (false negatives) represent misclassified samples.

In general, values approaching 1.0 indicate that the model achieves high overall classification accuracy (Accuracy), strong discriminative ability between positive and negative samples (ROC-AUC), a good balance between false positives and false negatives (F1), and robust, balanced predictive performance across all confusion matrix components (MCC). In addition, high recall reflects the model’s ability to correctly identify most positive samples; high precision indicates reliable positive predictions with low false-positive rates; and high specificity demonstrates effective recognition of negative samples.

#### 2.1.2. Ablation Study

**Figure 2:**
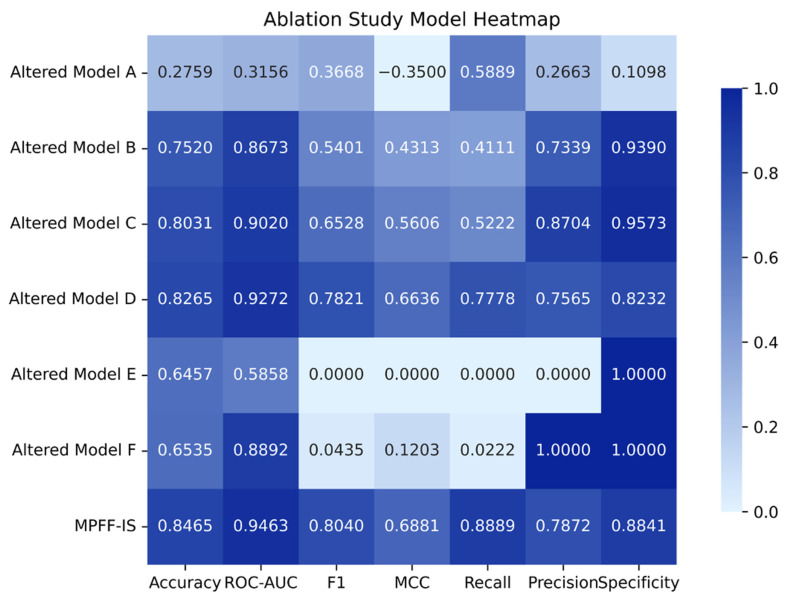
Model ablation study. MPFF-IS: Full model (MPNN|ESM2|Fusion|MLP), A: (FingerPrint|one-hot|cat|MultiHead), B: (MPNN|ESM2|Fusion|MultiHead), C: (MPNN|ESM2|cat|MLP), D: (MPNN|one-hot|Fusion|MLP), E: (FingerPrint|one-hot|cat|MLP), F: (FingerPrint|ESM2|cat|MLP).

To evaluate the contribution of each functional module, we constructed a series of comparative models by systematically replacing and recombining the compound representation method, protein-encoding strategy, feature-fusion approach, and prediction module. The performance of these model variants on the general dataset test set is illustrated in the heatmap in Figure 2.

Overall, models employing FingerPrint for compound representation and one-hot encoding for proteins (Altered Models A and E) performed relatively poorly, suggesting that traditional molecular fingerprints combined with simple sequence encoding have limited capacity to capture complex protein–ligand interactions in this task. Although Altered Model E achieved an accuracy of 0.6457, its F1 score and recall were both 0, indicating a pronounced class prediction bias.

When compounds were represented using MPNN and proteins were encoded using ESM2 (Altered Models B and C), overall performance improved significantly, indicating that deep representations can more effectively capture structural and sequence information. Among these, Altered Model C (cat + MLP) outperformed Altered Model B (Fusion + MultiHead), particularly in terms of F1 and MCC, suggesting that the MLP prediction head may be more stable than the MultiHead architecture for this task.

Altered Model D, which combines MPNN with one-hot protein encoding and employs Fusion + MLP, still achieved relatively strong performance. This indicates that, within the current framework, the fusion module and prediction head can partially compensate for the presence of less informative protein features. However, its overall performance remains slightly inferior to that of the final model.

The final model, MPFF-IS (MPNN + ESM2 + Fusion + MLP), achieved the best and most balanced performance (Accuracy 0.8465, ROC-AUC 0.9112, F1 0.8040, MCC 0.6881), demonstrating that the combination of MPNN and ESM2 representations, along with Fusion-based feature interaction and an MLP decoder, provides advantages for this task.

#### 2.1.3. Model-Based Screening

A total of 133,856 samples from the experimental dataset were fed into the pre-trained model for prediction, yielding 2663 compounds identified as highly reliable candidates. These compounds were subsequently subjected to molecular docking simulations to estimate their binding energies. Based on the docking results, compounds with lower binding energies were further selected for subsequent analysis to evaluate their stability.

### 2.2. Molecular Docking Simulation

#### 2.2.1. Vina Docking Analysis

The development strategies for KRAS G12D inhibitors are diverse, and different mechanisms correspond to distinct binding sites and conformational states. In this study, two representative inhibition strategies were selected for comparison:

(1) GDP-state stabilizing inhibitors—represented by MRTX1133, which selectively binds to the GDP-bound conformation of KRAS G12D and stabilizes the Switch II pocket. This stabilizes KRAS in its inactive state, thereby blocking GEF-mediated GDP/GTP exchange and inhibiting downstream signaling pathways [19].
(2) GDP/GTP-binding type inhibitors—represented by BI-2852, which binds at the interface between the Switch I and Switch II regions. It inhibits the signaling cascade by preventing the interaction between KRAS and RAF and can bind to the active state of KRAS G12D [31].

Based on this, two groups of in situ molecular docking experiments were performed on the 2663 screened candidate ligands. In the first group, MRTX1133 was used as the reference ligand (ref_lig), with the system composed of KRAS G12D + Mg^2+^ + GDP. In the second group, BI-2852 was used as the reference ligand, with the system composed of KRAS G12D + Mg^2+^ + GTP.

Docking was performed using AutoDock Vina, and the results were ranked in descending order by Vina scores. The top three ligands from each group were selected for subsequent molecular dynamics (MD) simulations.

**Figure 3:**
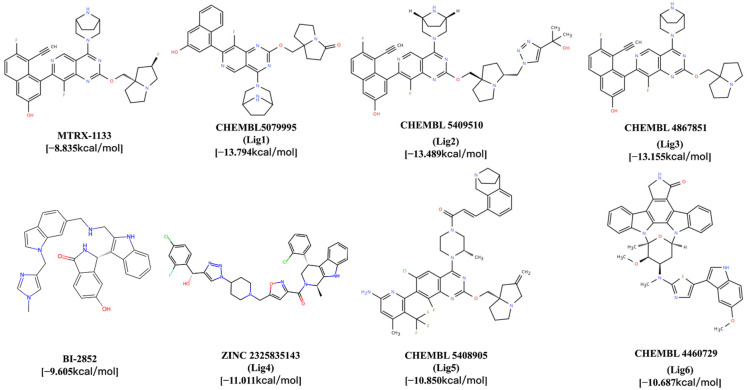
Structural diagrams of clinical compounds and candidate compounds. The top three ligands from each group (Lig1–Lig6) were selected for subsequent molecular dynamics (MD) simulations based on these superior docking scores.

For the GDP-state stabilizing inhibitors, the top three ligands were CHEMBL5079995 (−13.794 kcal/mol), CHEMBL5409510 (−13.489 kcal/mol), and CHEMBL4867851 (−13.155 kcal/mol), designated Lig1, Lig2, and Lig3, respectively. The structures of MRTX1133 and Lig1–Lig3 are presented in Figure 3. For the GDP/GTP-binding type inhibitors, the top three ligands were ZINC2325835143 (−11.011 kcal/mol), CHEMBL5408905 (−10.850 kcal/mol), and CHEMBL4460729 (−10.687 kcal/mol), which were designated as Lig4, Lig5, and Lig6, respectively. The structures of BI-2852 and Lig4–Lig6 are also shown in Figure 3.

In comparison, the Vina scores of the reference ligands MRTX1133 and BI-2852 were only −8.835 kcal/mol and −9.605 kcal/mol, respectively, indicating that the screened candidate compounds exhibit stronger binding affinity.

#### 2.2.2. Binding Mode Analysis

To further investigate the interaction characteristics between the candidate compounds and KRAS G12D, binding-mode analysis was performed on ligands with the top Vina scores. The results show that different ligands exhibit stable non-covalent interactions within both the Switch I and Switch II regions.

**Figure 4:**
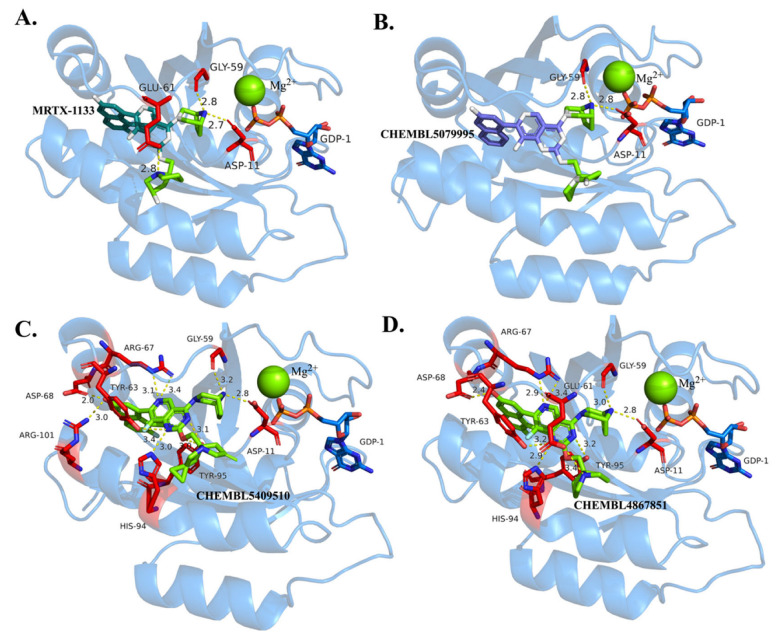
Atomic interactions between the KRAS G12D pocket and GDP-like inhibitors. (**A**) MRTX1133, (**B**) Lig 1, (**C**) Lig2, (**D**) Lig3.

In GDP-state stabilization inhibitors, as shown in Figure 4, all four ligands form well-defined hydrogen bond networks within the GDP-binding pocket. Based on the docking conformation (Figure 4A), MRTX1133 establishes stable hydrogen bonds with key residues, including Glu61 (~2.8 Å), Asp11 (~2.7 Å), and Gly59, along with coordination interactions with Mg^2+^, which collectively stabilize its binding conformation. These interactions define the core interaction pattern within the KRAS G12D binding pocket and are conserved among the screened candidate compounds.

For the candidate compounds, Lig1 (Figure 4B) largely preserves this core binding mode, forming hydrogen bonds with Glu61, Asp11, and Gly59, while maintaining coordination-related interactions with Mg^2+^. This indicates a high degree of consistency with the reference binding pattern. In particular, Lig1 forms hydrogen bonds of approximately 2.8 Å with Asp11 and Gly59 and maintains stable polar interactions with Glu61, collectively contributing to the initial anchoring of the ligand within the binding pocket.

Lig2 and Lig3 exhibit more extensive interaction patterns while retaining the core interactions described above. Specifically, Lig2 forms multiple hydrogen bonds with Asp68 (~2.0 Å), Arg67 (~3.1–3.4 Å), Tyr63 (~3.4 Å), and His94 (~3.0 Å), thereby establishing an extended hydrogen-bond network that bridges the Switch II region and adjacent loop structures. Similarly, Lig3 forms multiple hydrogen bonds with Asp68 (~2.4 Å), Tyr63 (~3.2 Å), His94 (~2.9 Å), and Tyr95 (~3.0–3.2 Å), further increasing the interaction density within the binding pocket.

In addition to hydrogen bonding, Lig2 and Lig3 exhibit pronounced hydrophobic interactions with residues such as Val7 and Leu56, which facilitate stable embedding of the ligands within the hydrophobic cavity and reduce conformational fluctuations. These hydrophobic interactions act synergistically with polar interactions to enhance overall binding stability.

Furthermore, the aromatic rings of Lig2 and Lig3 participate in strong π–cation interactions with Arg67, as well as π–π stacking interactions with aromatic residues such as Tyr63 and Tyr95. These interactions not only stabilize the aromatic moieties within the deep binding pocket but also increase local conformational rigidity, thereby enhancing protein–ligand binding affinity.

Overall, while preserving the core hydrogen bonds (Glu61, Asp11, and Gly59), Lig2 and Lig3 establish additional hydrogen bonds with residues such as Asp68, His94, and Tyr95, and further stabilize the complexes through hydrophobic and π-mediated interactions. This results in a more compact and stable interaction network compared to Lig1, suggesting enhanced binding stability and potential inhibitory activity.

It should be noted that residue numbering follows the KRAS sequence in the PDB structure (7RPZ). Due to the absence of N-terminal residues in this structure, numbering offsets may occur (e.g., Asp11 corresponds to Asp12 in the canonical sequence).

**Figure 5:**
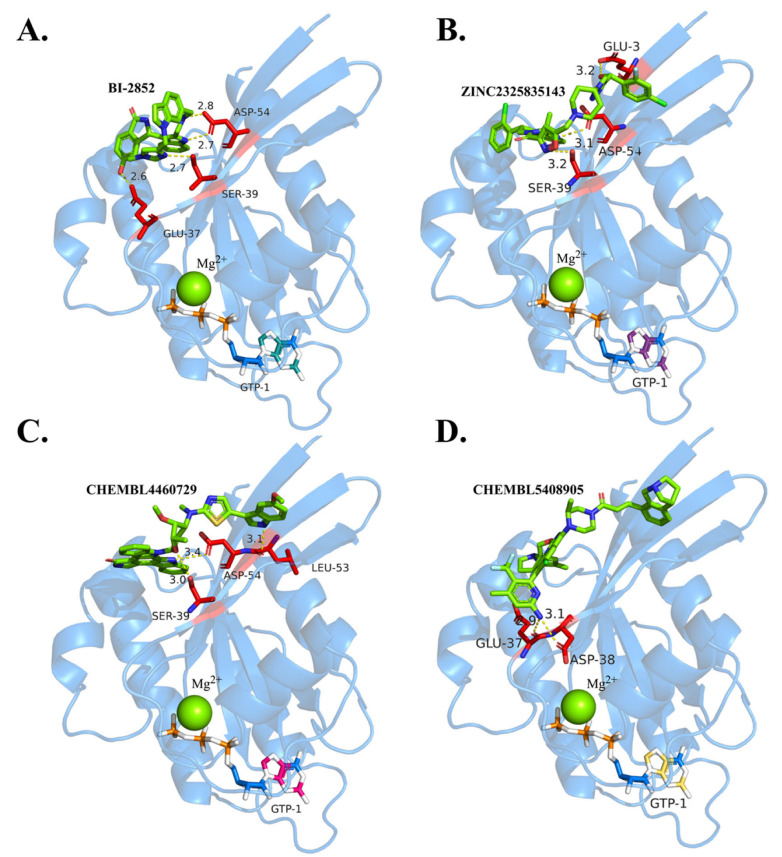
Atomic interactions between the KRAS G12D pocket and GDP/GTP-binding inhibitors. (**A**) BI2852, (**B**) Lig4, (**C**) Lig5, (**D**) Lig6.

Using the BI-2852 binding conformation as a reference, the binding modes of multiple ligands with KRAS G12D were comparatively analyzed, as shown in Figure 5. The results indicate that although the ligands exhibit slight positional variations within the binding pocket, they are all predominantly located around the open Switch I/II regions of KRAS G12D, forming stable hydrogen-bonding and electrostatic interactions. BI-2852 forms an extensive hydrogen-bonding network with polar residues such as Glu37, Ser39, and Asp54, thereby significantly contributing to its binding stability.

For the candidate compounds, Lig4, Lig5, and Lig6 also display well-defined interaction patterns within the KRAS G12D binding pocket, primarily involving hydrogen bonding, hydrophobic interactions, and, in some cases, salt-bridge formation. Lig4 exhibits a relatively simple interaction pattern but retains essential stabilizing contacts. It primarily forms a hydrogen bond with Ser39, serving as an anchoring point, while establishing hydrophobic contacts with residues such as Lys5, Leu52, Asp54, Leu56, Gln70, and Tyr71, thereby maintaining its stable positioning within the binding cavity.

Lig5 forms multiple hydrogen bonds with key residues, including Glu3, Asp54, and Leu53, thereby establishing a stable polar interaction network within the binding region. In addition, it engages in extensive hydrophobic interactions with residues such as Lys5, Val7, Arg41, Leu52, and Leu56, which facilitate its stable embedding within the hydrophobic pocket. Notably, the involvement of charged residues such as Lys42 and Asp54 further enhances electrostatic complementarity within the system.

Lig6 exhibits a similar but more structurally organized interaction pattern. It forms hydrogen bonds with Glu37 and Asp38, indicating effective polar recognition within the Switch I/II regions. More importantly, Lig6 forms a salt bridge with Asp54, thereby significantly strengthening electrostatic stabilization. Additionally, hydrophobic interactions with residues such as Arg41 and Leu52 further stabilize the ligand within the binding pocket.

Overall, these key residues play important roles in structural recognition across different ligand-binding modes. In particular, Asp54 and Ser39 recur across multiple interaction patterns, suggesting that they may serve as core anchoring sites for KRAS G12D inhibitor binding and provide important guidance for subsequent structural optimization.

All binding mode analyses were performed using the Protein–Ligand Interaction Profiler (PLIP, 2025 version) as described by Schake and Bolz et al. Default parameters were applied throughout the analysis to ensure consistency and reproducibility in the identification of interactions.

### 2.3. Molecular Dynamics Simulation

Molecular dynamics (MD) simulations were performed on the KRAS G12D + Mg^2+^ + GDP/GTP + ligand systems. The root-mean-square deviation (RMSD) was calculated to characterize conformational changes in the protein–ligand complexes and the flexibility of amino acid residues during the simulation. The root-mean-square fluctuation (RMSF) was used to quantify the positional deviations of specific atoms or residues relative to a reference structure. The radius of gyration (Rg) reflects the system’s compactness throughout the MD simulation.

#### 2.3.1. GDP-State Stabilizing Inhibitors

**Figure 6:**
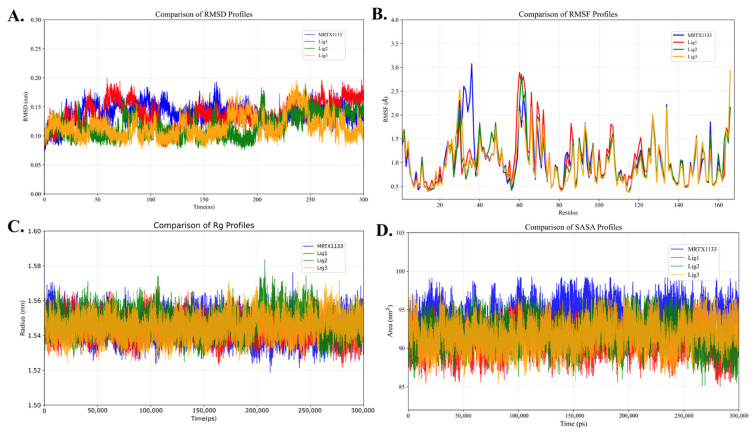
MD simulations of the KRAS G12D protein bound to GDP-like inhibitors (targeting the inactive state). The stability and flexibility of the complexes are assessed using: (**A**) RMSD (structural stability), (**B**) RMSF (residue flexibility), (**C**) Rg (system compactness), and (**D**) SASA (solvent exposure).

Figure 6 presents the time evolution of RMSD, RMSF, Rg, and SASA for the KRAS G12D + Mg^2+^ + GDP + ligand complexes. As shown in Figure 6A, all four systems undergo an initial conformational adjustment phase (0–50 ns), followed by stabilization. During the 100–200 ns interval, the RMSD values for all systems remain relatively low, indicating overall structural stability of the complexes.

Specifically, the RMSD values of Lig2 and Lig3 stabilize at approximately 0.10 nm, while those of MRTX1133 and Lig1 are around 0.15 nm. It should be noted that this difference of approximately 0.05 nm falls within the typical fluctuation range of molecular dynamics simulations; therefore, no significant differences in stability are observed among the systems. Within the 150–200 ns interval, Lig2 and Lig3 exhibit slight fluctuations; however, the variation remains small (approximately 0.05 nm) and within the noise range, suggesting that no significant conformational drift occurs during the simulation.

RMSF analysis (Figure 6B) shows that fluctuations in all complexes are mainly localized in Switch I (residues 26–38), Switch II (residues 57–72), and the GDP-binding region (around residue 169), indicating that these regions are more flexible than other structural domains. Notably, compared with the MRTX1133–KRAS G12D complex, Lig2 and Lig3 exhibit reduced RMSFs in the Switch I region, suggesting the formation of more stable binding conformations within this pocket.

This observation is consistent with the interaction analysis results: Lig2 and Lig3 form stable hydrogen-bond networks with key residues such as Asp68, His94, and Tyr95, thereby enhancing local structural stability and the binding rigidity of the complexes.

From the Rg profiles (Figure 6C), all four complexes exhibit nearly identical values of approximately 1.55 nm, indicating that the overall compactness of the systems is well maintained throughout the simulation, without significant structural expansion or collapse. The SASA curves (Figure 6D) further reveal that the MRTX1133–KRAS G12D complex exhibits slightly higher SASA values than the other systems, suggesting greater solvent exposure. In contrast, the complexes with Lig2 and Lig3 exhibit lower SASA values, indicating more compact binding conformations and enhanced structural stability.

In summary, although RMSD analysis does not reveal significant differences, the complexes formed by Lig2 and Lig3 with KRAS G12D exhibit relatively improved structural stability, tighter binding, and more favorable interactions with key residues, suggesting their potential for higher binding affinity and biological stability.

#### 2.3.2. GDP/GTP-Binding Type Inhibitors

**Figure 7:**
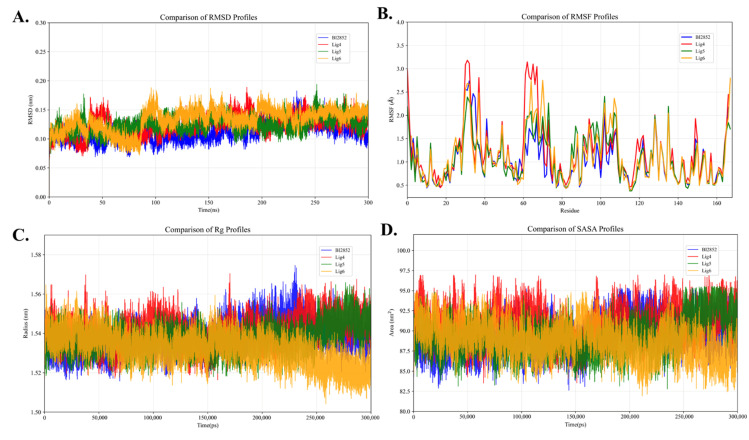
MD simulations of the KRAS G12D protein bound to GDP/GTP-binding inhibitors (targeting the active state). The stability and flexibility of the complexes are assessed using: (**A**) RMSD (structural stability), (**B**) RMSF (residue flexibility), (**C**) Rg (system compactness), and (**D**) SASA (solvent exposure).

Figure 7 presents the time evolution of RMSD, RMSF, Rg, and SASA for the KRAS G12D–Mg^2+^–GTP–ligand complexes during 300 ns molecular dynamics simulations. The RMSD curves of all four ligand-bound complexes exhibit an initial fluctuation characterized by an increase followed by a decrease within the first 100 ns, after which they gradually stabilize. The final RMSD values remain around 0.13 nm, indicating that the systems reach equilibrium and adopt relatively compact, stable conformations. 

The RMSF results (Figure 7B) show pronounced fluctuations in the Switch I region (residues 26–38), Switch II region (residues 57–72), and the GTP-binding region (around residue 169), suggesting that these regions retain relatively high flexibility after ligand binding. Notably, the three candidate ligands exhibit larger fluctuations in the Switch II region compared to the reference compound BI-2852, indicating that they may interfere with the Switch II conformation and thereby hinder the binding of other molecules to KRAS G12D. Interestingly, Lig5 shows significantly reduced fluctuations around residue 168, suggesting the formation of more stable interactions in the GTP-binding region, thereby enhancing the overall stability of the complex.

Figure 7C shows the radius of gyration (Rg) profiles of the complexes. The results indicate that BI-2852, Lig4, and Lig5 maintain stable Rg values of approximately 1.53 nm throughout the simulation, suggesting that their overall structures remain compact. In contrast, Lig6 exhibits a noticeable decrease in Rg around 230 ns, reaching approximately 1.52 nm, indicating further conformational compaction. A slight increase follows this, but the system remains relatively stable overall.

Figure 7D illustrates the solvent-accessible surface area (SASA) changes. The SASA values of BI-2852, Lig4, and Lig5 remain relatively constant at approximately 90 nm^2^, indicating stable interactions with the solvent. In contrast, Lig6 shows a slight increase in SASA around 150 ns, followed by a decrease to approximately 86 nm^2^ at 210 ns, and then returns to around 90 nm^2^ at 250 ns before another brief decline, reflecting a degree of dynamic fluctuation.

Overall, all four ligand–KRAS G12D–Mg^2+^–GTP complexes exhibit good structural stability over the long-timescale simulations. Among them, Lig5 demonstrates relatively stronger local stability in the GTP-binding region. At the same time, Lig6 shows a tendency toward conformational tightening in the later stages of the simulation, suggesting that its interaction with KRAS G12D may involve a distinct conformational modulation mechanism. These findings provide a reliable dynamic basis for subsequent binding free-energy calculations and binding-mode analyses.

### 2.4. MMPBSA Analysis

#### 2.4.1. GDP-State Stabilizing Inhibitors

To investigate the binding stability and potential inhibitory mechanisms of different ligands toward the KRAS G12D–GDP–Mg^2+^ complex, binding free energies were calculated using the MMPBSA method based on 300 ns molecular dynamics trajectories. The Poisson–Boltzmann (PB) solvent model was employed, and the energy components included electrostatic interactions (ΔE_EL), van der Waals interactions (ΔE_VDW), as well as polar and nonpolar solvation energies (ΔE_PB and ΔE_NPOLAR). The results are summarized in Table 2.

The calculated binding free energies of the selected candidate ligands with the KRAS G12D–GDP–Mg^2+^ complex were −41.92 kcal·mol^−1^ (Lig1), −53.61 kcal·mol^−1^ (Lig2), and −54.34 kcal·mol^−1^ (Lig3), indicating relatively strong binding affinities. Among them, Lig3 exhibits the lowest binding free energy, suggesting the most favorable binding stability, followed by Lig2. Notably, the binding free energies of the candidate ligands are overall comparable to that of the known inhibitor MRTX1133 (ΔG_bind = −54.91 kcal·mol^−1^), indicating that the screened compounds possess binding capabilities similar to the reference inhibitor.

Further analysis of energy decomposition reveals that all ligands exhibit significant contributions from both van der Waals and electrostatic interactions. For MRTX1133, the van der Waals interaction (ΔE_VDW = −69.60 kcal·mol^−1^) and the electrostatic interaction (ΔE_EL = −40.06 kcal·mol^−1^) are both substantial, contributing critically to its stability. Among the candidate ligands, Lig3 shows the strongest van der Waals interaction (ΔE_VDW = −71.31 kcal·mol^−1^), followed by Lig2 (ΔE_VDW = −67.11 kcal·mol^−1^). Although the magnitudes of van der Waals and electrostatic interactions vary slightly among the candidate ligands, they consistently maintain favorable interaction profiles.

At the same time, all ligands exhibit a certain degree of polar solvation energy penalty (positive ΔE_PB values). The solvation energies of the candidate ligands are comparable to that of MRTX1133, suggesting that similar levels of solvent shielding effects must be overcome in aqueous environments. While this solvation penalty partially offsets favorable interactions, it does not substantially alter the overall binding trends.

Mechanistically, these candidate ligands form stable interactions with the KRAS G12D active pocket through electrostatic and van der Waals forces, which may help stabilize the GDP-bound conformation and influence downstream signaling processes. In addition, strong nonpolar interactions facilitate hydrophobic stacking, further enhancing the overall stability of the complexes.

In summary, Lig3 and Lig2 exhibit binding free energies comparable to that of the known inhibitor MRTX1133, indicating strong binding potential and warranting further structural optimization and experimental validation, whereas Lig1 shows relatively weaker binding capability. These findings provide important theoretical support for the design of lead compounds targeting KRAS G12D.

#### 2.4.2. GDP/GTP-Binding Type Inhibitors

In addition, we investigated the binding stability and potential inhibitory mechanisms of different ligands toward the KRAS G12D–Mg^2+^ complex, as summarized in Table 3. Since the current simulation system does not include GDP, this analysis primarily focuses on evaluating the relative binding affinities of candidate ligands within the apo (empty-pocket) state of KRAS G12D.

The calculated results indicate that the binding free energies of the three candidate ligands are comparable to that of the reference compound BI-2852. Among them, Lig5 exhibits the lowest binding free energy (ΔG_bind = −30.00 kcal·mol^−1^), followed by Lig4 (−29.83 kcal·mol^−1^), while Lig6 shows a relatively higher value (−16.27 kcal·mol^−1^). Overall, Lig5 and Lig4 appear slightly more favorable than BI-2852 (−25.07 kcal·mol^−1^); however, this difference of approximately 4–5 kcal·mol^−1^ falls within the typical error range of the MM/PBSA method. Therefore, these results are more indicative of comparable binding capabilities rather than a significant advantage.

Energy decomposition analysis reveals that van der Waals and electrostatic interactions primarily drive ligand binding. In particular, Lig5 exhibits strong favorable contributions from both van der Waals interactions (ΔE_VDW = −48.93 kcal·mol^−1^) and electrostatic interactions (ΔE_EL = −20.01 kcal·mol^−1^), which largely account for its relatively strong binding affinity. In comparison, BI-2852 shows a weaker van der Waals contribution (ΔE_VDW = −35.09 kcal·mol^−1^), while Lig4 and Lig6 exhibit van der Waals energies of −49.71 kcal·mol^−1^ and −26.08 kcal·mol^−1^, respectively, reflecting varying degrees of hydrophobic contribution.

Notably, all ligands exhibit significant polar solvation energy penalties (positive ΔE_PB values). Among them, Lig5 shows a relatively high polar solvation energy (ΔE_PB = +43.81 kcal·mol^−1^), indicating that stronger polar interactions require overcoming a larger solvation penalty. Nevertheless, the net contribution of electrostatic interaction and solvation energy (ΔE_EL + ΔE_PB = +23.80 kcal·mol^−1^) still plays a role in determining the overall binding free energy, suggesting a “high compensation–high penalty” characteristic of electrostatic interactions in this system.

In contrast, although Lig6 demonstrates moderate van der Waals interactions, its overall binding free energy is significantly less favorable (−16.27 kcal·mol^−1^), primarily due to weaker electrostatic interactions and insufficient electrostatic complementarity, which limit its binding stability.

In summary, within the KRAS G12D system, Lig5 and Lig4 exhibit binding free energies comparable to that of BI-2852 and show potential for further optimization. Lig5 demonstrates the most favorable binding trend due to the combined contributions of multiple energy components, whereas Lig6 exhibits relatively weaker binding capability. These findings suggest that different ligands can achieve stable binding to KRAS G12D through distinct combinations of van der Waals and electrostatic interactions, providing valuable insights for subsequent lead optimization and rational drug design.

### 2.5. Principal Component Analysis

**Figure 8:**
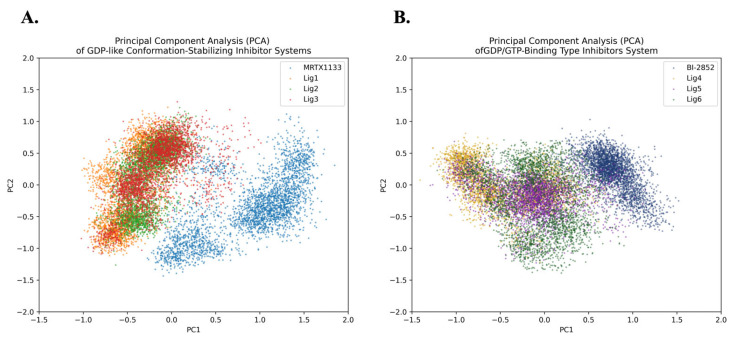
Principal component analysis. (**A**) GDP-like Conformation-Stabilizing Inhibitor Systems; (**B**) GDP/GTP-Binding Type Inhibitor Systems.

The results of principal component analysis (PCA), as shown in Figure 8, indicate that the conformational space of the reference inhibitor MRTX1133 differs overall from those of the candidate ligand systems, although partial overlap remains. It should be noted that the PCA in this study is based on the Cα atoms of the protein; therefore, the results primarily reflect changes in protein conformational dynamics under different ligand-bound states, rather than the intrinsic conformational properties of the ligands themselves.

Notably, the protein conformational distributions for the candidate compounds Lig1–Lig3 are relatively close to one another and exhibit some overlap with the reference system. This suggests that these compounds can induce protein conformational states similar to those associated with a known active inhibitor upon binding. This observation indirectly indicates that the constructed screening model exhibits some selectivity, enabling it to preferentially identify compounds that may exert similar conformational regulatory effects on the protein.

At the same time, the candidate ligand systems still exhibit a degree of conformational deviation relative to the reference inhibitor, suggesting that, while key functional features are preserved, these ligands may modulate protein dynamics to varying degrees. This “similar yet not identical” conformational distribution pattern may provide additional opportunities for further optimization in terms of binding stability and drug-like properties.

### 2.6. Comparative Analysis of the Structural Features and Mechanisms of Action of Candidate Compounds and Known KRAS G12d Inhibitors

At the level of structural characteristics and mechanisms of action, a comparison between the candidate compounds identified in this study (CHEMBL5079995, CHEMBL5409510, CHEMBL4867851, ZINC2325835143, CHEMBL5408905, and CHEMBL4460729) and previously reported KRAS G12D inhibitors reveals notable similarities in key structural features and binding modes, while also demonstrating a degree of structural optimization. In previous studies, for example, the representative natural product inhibitor NPC489264 identified by Nadee et al. [28] has a molecular weight of approximately 494 Da and a relatively compact structure. It primarily binds through a hydrogen-bond network within the Switch I/II pocket (e.g., interactions with Asp12, Glu62, and Arg68), accompanied by limited hydrophobic interactions, with its mechanism largely dominated by polar interactions. Similarly, candidate compounds such as ZINC05524764, identified by Ajmal et al. [26] using traditional machine learning approaches, tend to be small in molecular size, relatively polar, and reliant on hydrogen bonding and electrostatic interactions.

In contrast, the compounds identified in this study generally exhibit higher molecular weights and more complex scaffold architectures. Representative compounds such as CHEMBL5409510 and CHEMBL4867851 contain polycyclic aromatic systems and nitrogen-containing heterocycles, connected via flexible linkers to form a structural pattern characterized by a “rigid aromatic core + flexible linker + polar terminal groups.” This structural feature is consistent with the overall architecture of the known high-affinity inhibitor MRTX1133, but with enhanced spatial occupancy, enabling more effective filling of the Switch II pocket and the GDP-binding region. At the interaction level, the candidate compounds in this study not only form stable hydrogen bonds with key residues such as Glu61, Asp11/12, Gly5, Glu37, Ser39, and Asp54, but also exhibit stronger hydrophobic interactions and π–π/π–cation interactions (e.g., with Arg67/Arg68). Notably, CHEMBL5409510 and CHEMBL4867851 display characteristic aromatic ring–cation interactions, which are less commonly observed in previously reported small-molecule inhibitors.

From a mechanistic perspective, natural product-like compounds such as NPC489264 primarily achieve selective binding to KRAS G12D through a “hydrogen bond-driven + local polarity recognition” mode, particularly via specific hydrogen bonding with the mutated residue Asp12. In contrast, the compounds identified in this study further incorporate a synergistic mechanism of “hydrophobic packing + π-system stabilization.” These compounds not only recognize key polar residues but also enhance cavity occupancy through larger molecular scaffolds, thereby improving overall complex stability and binding free energy. This trend is also supported by docking and molecular dynamics results, in which the candidate compounds identified in this study show binding affinities and structural stability comparable to or better than those of reference inhibitors (e.g., MRTX1133 and BI-2852), rather than relying solely on hydrogen-bond networks.

Furthermore, regarding targeting mechanisms, while the candidate compounds in this study, NPC489264 and MRTX1133, primarily act on the Switch I/II region, the compounds identified here exhibit greater occupancy within the Switch II pocket and adjacent hydrophobic regions. This suggests a potentially stronger capability to stabilize the inactive conformation of KRAS or to interfere with effector protein binding. Overall, the compounds identified in this study retain the key binding characteristics of known KRAS G12D inhibitors while further optimizing interaction patterns through enhanced hydrophobic interactions and spatial occupancy, demonstrating improved structural rationality and potential druggability.

## 3. Materials and Methods

### 3.1. Dataset Construction

Training Dataset

To systematically evaluate model performance, positive protein–compound pairs were collected from the BindingDB bioactivity database (data retrieval time: March 2025). The screening criterion was defined as compounds with IC_50_ values ≤ 6.1 nM against the target protein, based on the activity threshold of the PDAC inhibitor MRTX1133 [32]. Samples with IC_50_ values ≥ 6.1 nM were defined as negative samples. After deduplication and filtering, a total of 2540 data points were obtained.

To avoid information leakage from identical or highly similar chemical scaffolds distributed across different subsets, the dataset was partitioned based on Bemis–Murcko scaffolds. Specifically, samples were first grouped by molecular scaffold, and a constrained, optimization-based greedy strategy was then used to assign entire scaffold groups to the training, validation, and test sets. This approach aims to approximate an 8:1:1 split while minimizing deviations in label distribution between each subset and the overall dataset [33]. Such a scaffold-based splitting strategy preserves structural diversity across subsets while maintaining balanced dataset proportions, thereby improving the reliability of model evaluation and generalization.

Experimental Dataset

To construct a candidate compound library for virtual screening and activity evaluation, small-molecule data were systematically collected from the IBS, NPASS, ChEMBL, and ZINC public databases (citation). During data analysis, we observed that KRAS G12D inhibitors often deviate from Lipinski’s rules, particularly regarding higher molecular weight and lower solubility [19,34,35]. To improve the drug-likeness and rationality of candidate selection, we defined filtering criteria based on the physicochemical property distributions of known KRAS G12D inhibitors. Specifically, after removing the top and bottom 10% of extreme values, the following thresholds were applied: molecular weight (MW) 550–1500, partition coefficient (LogP) 4–8, hydrogen-bond donors (HBD) 1–4, and hydrogen-bond acceptors (HBA) 7–13. These thresholds were determined by excluding the top and bottom 5% outliers for each descriptor to reduce the influence of extreme values on the screening results. Furthermore, duplicate compounds were identified and removed based on canonical SMILES to ensure the uniqueness of the screening library.

After applying these criteria, the number of retained compounds from each database was as follows: IBS (7332), NPASS (2694), ChEMBL (68,350), and ZINC (55,480). In total, 133,856 compounds meeting the selection criteria were obtained, forming the dataset for subsequent virtual screening and activity validation.

### 3.2. Model Construction

**Figure 9:**
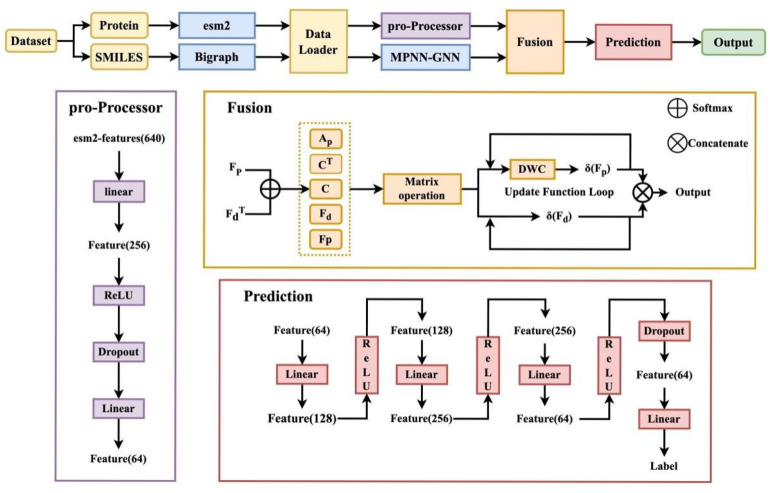
Schematic diagram of the model workflow.

The architecture of the proposed model is illustrated in Figure 9. It consists of four main components: a target protein feature extraction module, a compound feature extraction module, a feature fusion module, and an activity prediction module. This model integrates the ESM-2 protein feature extractor with an MPNN-based graph neural network to represent compounds, enabling effective modeling of ligand–target interactions through feature fusion.

#### 3.2.1. Target Protein Feature Extraction

Protein representations were extracted using the pre-trained ESM-2 model (esm2_t33_650M_UR50D) [36], which captures rich sequence-level evolutionary and structural information from large-scale protein datasets. Given an amino acid sequence of length L, the model outputs residue-wise embeddings of dimension L × 1280 from the final hidden layer. To obtain a fixed-length protein representation, mean pooling was applied across all residue embeddings, enabling global contextual aggregation while preserving sequence-level semantics.

To align with compound feature representations, the protein embeddings were further transformed through a multi-layer projection module. Specifically, a fully connected layer first reduced the dimensionality from 1280 to 256, followed by a ReLU activation and dropout regularization to enhance nonlinearity and prevent overfitting. The features were subsequently projected to a 64-dimensional space, ensuring both dimensional consistency with compound features and retention of essential semantic information.

For efficient batch processing, a PyTorch(v2.6.0) DataLoader was used with a custom collate fn to handle heterogeneous data types. Molecular graph data were batched using DGL’s graph batching mechanism, where node and edge features were concatenated along the feature dimension. Protein sequences were padded to a uniform length within each batch to enable parallel computation, and adjacency matrices were zero-padded to a consistent size before stacking. This unified batching strategy ensures compatibility between graph-structured molecular data and sequence-based protein representations within the model.

#### 3.2.2. Compound Feature Extraction

Compound molecules are converted into graph representations using the DGL-LifeSci toolkit [37], where atom-level features (74 dimensions) and bond-level features (12 dimensions) are extracted. The adjacency matrix, representing atomic connectivity, is computed using RDKit. A compound feature extractor is then constructed based on the Message Passing Neural Network (MPNN) framework [38,39].

MPNN is a classical graph neural network (GNN) architecture widely used for graph-structured data tasks, such as molecular modeling. Its core idea is to aggregate and update local node information using a message-passing paradigm, thereby learning higher-order representations of each node within the graph. The model typically consists of two main phases:

(1) Message Passing Phase:

In each graph convolutional layer, a node receives information from its neighbors. Specifically, for node i, messages from its neighbors are computed based on both node features and edge features, and then aggregated as:

$$m_{i}^{\left(l\right)}=\sum_{j\in N(i)}f_{\Theta }(h_{i}^{\left(l\right)},h_{j}^{\left(l\right)},e_{ij})$$

where $h_{i}^{\left(l\right)}$ and $h_{j}^{\left(l\right)}$ denote the feature representations of nodes i and j at layer l, $e_{ij}$ represents the edge feature between nodes i and j, and $f_{\Theta }$ is a learnable message function parameterized by θ. This formulation enables the model to incorporate both local structural information and chemical bond characteristics during feature propagation.

(2) Node Update Phase:

After receiving the aggregated messages, the target node updates its representation by integrating its current state with the aggregated information via an update function. The update process is as follows:

$$h_{i}^{\left(l+1\right)}=\sigma (W\cdot [h_{i}^{\left(l\right)}∥m_{i}^{\left(l\right)}])$$

This update mechanism enables the model to continuously integrate its own information with the neighborhood context during multi-layer propagation, thereby capturing higher-order structural dependencies in the molecular graph.

In this model, the node feature update process begins with a linear transformation of the initial node features, followed by a nonlinear activation function to enhance representational capacity. Subsequently, six rounds of message passing and neighbor aggregation are performed on the graph, enabling the model to progressively capture broader structural context and iteratively refine node representations.

The node update mechanism in each graph convolution layer can be formally expressed as follows:

$$h_{i}^{l+1}=h_{i}^{l}+\sum_{j\in N\left(i\right)}\left(\left\{f_{\Theta }\left(e_{ij}\right)\cdot h_{j}^{l},j\in N\left(i\right)\right\}\right)$$

After six rounds of message passing, the comprehensive features across the entire molecular graph are aggregated, yielding a final 64-dimensional feature vector for each compound.

#### 3.2.3. Feature Fusion

Following the Hybrid-GNN approach [40], we construct a comprehensive interaction model by integrating features from the protein and the compound. Given the protein feature matrix $F_{p}\in R^{L_{p}\times C_{p}^{s}}$ and the compound graph features $F_{d}\in R^{L_{d}\times C_{d}^{s}}$ with their adjacency matrix $A_{d}\in R^{L_{d}\times L_{d}}$, the first step is to establish an association matrix *C* by multiplying the two feature matrices $C=Softmax(F_{p}F_{d}^{T})\in R^{L_{p}\times L_{d}}$.

Softmax transforms a matrix into a probability distribution, calculated using the following formula:

$$\sigma (z_{i})=\frac{e^{z_{i}}}{\sum_{j=1}^{n}e^{z_{j}}}$$

Matrix *C* quantifies potential interactions between the protein’s amino acids and the compound’s atoms. The entire interaction system is then formally represented using a unified GNN computation formula, treating the protein–drug interaction as bridging connections between two sub-graphs:

$$F_{s}=[\begin{array}{cc} A_{p} & C\\ C^{T} & A_{d} \end{array}][\begin{array}{c} F_{p}\\ F_{d} \end{array}]$$

Here, $A_{p}$ represents the hypothetical adjacency matrix for the protein. Since a protein sequence does not inherently form a static atom-level graph, the term $A_{p}F_{p}$ is practically implemented using One-Dimensional Deep-Wise Convolution (DWC) on $F_{p}$, which effectively captures relationships between adjacent amino acids using a 3 ×1 convolutional kernel $A_{p}F_{p}=DWC(F_{p})$. This operation integrates the graph information of both the compound and the protein via the cross-coupling matrix *C*.

Feature Update and Final Representation Based on the unified GNN structure, the feature update functions for the protein and compound in each iteration are defined to facilitate the transfer of interaction information:

$$\begin{array}{c} \delta (F_{p})=DWC(\omega (F_{p}))+C\omega (F_{d})+F_{p}\\ \delta (F_{d})=C^{T}\omega (F_{p})+A_{d}\omega (F_{d})+F_{d} \end{array}$$

In these equations, ω represents a linear transformation followed by a nonlinear activation. The terms $C\omega (F_{d})$ and $C^{T}\omega (F_{p})$ are crucial, as *C* and *C*^T^ are applied to the compound and protein features, respectively, enabling the transfer of interaction information across the two entities. After *N* rounds of GNN iterations, the refined protein features $\delta (F_{p})$ and compound features $\delta (F_{d})$ are obtained. These are then concatenated to form the final structural feature representation, $F_{s}=Concatenate(\delta (F_{p}),\delta (F_{d}))$ which is used for downstream prediction of biological activity.

#### 3.2.4. Activity Prediction Module

A multilayer feedforward neural network is employed as the prediction module, with the architecture defined as: 64-dimensional input → 128-dimensional (ReLU) → 256-dimensional (ReLU) → 64-dimensional (ReLU + Dropout) → 1-dimensional output. This network performs nonlinear transformations on the fused features to predict activity.

#### 3.2.5. Training Strategy

To ensure effective and robust model training, systematic designs were implemented for the optimizer, loss function, and training strategy. The AdamW optimizer is adopted, which decouples weight decay from gradient updates, thereby improving training stability. The loss function is BCEWithLogitsLoss, integrating Sigmoid activation with binary cross-entropy loss. To prevent overfitting, an early stopping mechanism is introduced: training is terminated when the validation loss fails to improve for 20 consecutive epochs. Hyperparameter optimization is conducted using Bayesian optimization based on Gaussian processes, with a two-stage search strategy (coarse search followed by fine-tuning) to determine the optimal hyperparameter configuration.

Regarding model architecture parameters, the MPNN module performs multiple rounds (six iterations) of message passing to effectively capture both local and global structural information of molecular graphs. For protein representation, residue-level embeddings from the final layer of the ESM2 model (esm2_t33_650M_UR50D) are used as sequence features (dimension: 1280). In practice, embedding vectors are extracted for each amino acid residue and projected into a lower-dimensional feature space using a fully connected layer to enhance the representation of functionally relevant sequence information. During training, the parameters of ESM2 are kept frozen by default, with only the downstream projection layers and fusion module trained, thereby reducing computational cost and improving model stability. Additionally, dropout (rate = 0.3) is applied in both the feature projection layer and the prediction module to mitigate overfitting further.

### 3.3. Virtual Validation and Molecular Dynamics Simulation

#### 3.3.1. Molecular Docking

In this study, molecular docking was employed to screen potential lead compounds targeting human KRAS mutant proteins, particularly the G12D variant. Candidate compounds were first obtained through prior machine-learning-based screening and subsequently subjected to docking simulations against the KRAS G12D mutant protein using AutoDock Vina 1.2.0. AutoDock Vina incorporates an improved scoring function and enhanced search algorithms, enabling higher docking accuracy while maintaining computational efficiency [41,42]. The three-dimensional structures of KRAS G12D were obtained from the RCSB PDB database (PDB IDs: 7RPZ and 6GJ8). Protein structures were preprocessed by removing water molecules, adding polar hydrogens, and assigning Gasteiger charges before docking. Ionizable residues were assigned standard protonation states corresponding to physiological pH (7.4), ensuring chemically reasonable structures for docking.

The selection criteria for candidate compounds mainly include the following aspects:

(1) Binding energy (ΔG_binding): Lower binding energy indicates more stable interactions between the compound and the target protein;
(2) Interactions between ligands and KRAS residues: including hydrogen bonds, hydrophobic interactions, and electrostatic interactions [28].

In docking, AutoDockTools (ADT) was first used to convert the protein and ligand files into PDBQT format. Rigid-receptor docking was performed using AutoDock Vina 1.2.0, where the protein backbone and side chains were kept fixed while the ligands maintained full torsional flexibility. To define the search space, the grid box was centered on the native binding site of the co-crystallized ligand 6IC(MRTX1133) within the 7RPZ structure. The grid box was defined based on the known ligand-binding site, covering the Switch I/II regions and the GDP-binding pocket. The grid box coordinates, dimensions, and other docking parameters were set according to previous studies [34]. The specific center coordinates were set to (1.714, 4.927, −23.164), with grid box dimensions of 20 Å × 20 Å × 20 Å. This volume was sufficient to encompass the Switch I/II regions and the GDP-binding pocket, ensuring a comprehensive search of the target site. The exhaustiveness parameter was set to 8 to ensure thorough sampling of the ligand conformations.

Docking results were visualized and analyzed in PyMOL (v3.0.0), with key interaction patterns, including hydrogen bonding, hydrophobic interactions, and electrostatic interactions, evaluated. The binding energy calculated by AutoDock Vina can be approximately expressed as follows:

$$G_{\mathrm{binding}\;}E_{vdw}+E_{\mathrm{electrostatic}\;}+E_{hbond}+E_{\mathrm{hydrophobic}\;}+E_{\mathrm{entropy}\;}$$

#### 3.3.2. Molecular Dynamics Simulation Validation

To further evaluate the binding stability between candidate lead compounds and the KRAS G12D mutant, as well as the conformational dynamics of the complexes under physiological conditions, molecular dynamics (MD) simulations were performed using the GROMACS software package. This approach enables the assessment of ligand–target interactions over time and provides theoretical support for subsequent optimization of pharmacological properties and structural modification.

System Construction

The AMBER99SB-ILDN force field was applied to parameterize the protein system [31], and a dodecahedral solvent box was constructed using the TIP3P water model, ensuring a minimum distance of 1.0 nm between the box boundary and the protein surface. Na^+^ and Cl^−^ ions were added to neutralize the system and adjust the ionic concentration to approximately 0.15 mol/L, thereby mimicking physiological electrolyte conditions.

Energy Minimization and System Equilibration

Before simulation, the system underwent a two-stage energy minimization process. An initial energy optimization was performed, followed by 5000 steps of conjugate gradient minimization to eliminate steric clashes and unfavorable conformations. After energy minimization, the system was first equilibrated under the NVT ensemble to stabilize the temperature, followed by NPT equilibration to adjust the system pressure and density. This sequential protocol follows standard GROMACS simulation procedures to ensure proper system equilibration before production runs. The system temperature was gradually increased from 0 K to 150 K and then stabilized at 300 K, while the pressure was maintained at 1 atm, thereby ensuring physiologically relevant simulation conditions [30].

Production MD Simulation

After equilibration, the production phase was initiated. A time step of 2 fs was employed, and each complex system was simulated for a total duration of 300 ns [43], enabling characterization of its dynamic behavior in a solvent environment.

#### 3.3.3. Molecular Dynamics Analysis and Thermodynamic Calculation

To quantitatively evaluate the binding affinity and thermodynamic stability between the lead compounds and the KRAS G12D mutant, binding free energies of the protein–ligand complexes were estimated using the Molecular Mechanics/Poisson–Boltzmann Surface Area (MM/PBSA) method based on MD trajectories. MM/PBSA is a widely used and reliable approach for predicting binding affinities, as it extracts energetic contributions from simulation trajectories to assess thermodynamic stability.

In this study, the gmx_MMPBSA module in GROMACS was used to calculate binding free energies. Based on the equilibrated trajectory, one frame was extracted every 2 ns from the 300 ns MD simulation, yielding 150 frames for statistical energy analysis. The binding free energy (ΔG_bind) was calculated according to the following equation:

$$\Delta G_{\mathrm{bind}\;}=G_{\mathrm{complex}\;}-G_{\mathrm{receptor}\;}-G_{\mathrm{ligand}\;}\;G=E_{MM}+G_{\mathrm{polar}\;}+G_{\mathrm{nonpolar}\;}$$

where

E_gas represents the gas-phase energy, including van der Waals and electrostatic interactions;
G_sol denotes the solvation free energy, consisting of polar (PB) and nonpolar contributions;
G_nonpolar represents the nonpolar solvation free energy.

Specifically, E_gas includes bonded interactions (bond, angle, and dihedral terms) as well as non-bonded interactions (van der Waals and electrostatic terms). The polar solvation energy is calculated using the Poisson–Boltzmann (PB) equation, while the nonpolar solvation energy is estimated based on an empirical model related to the solvent-accessible surface area (SASA). The resulting binding free energy reflects the thermodynamic stability of complex formation and provides a quantitative basis for subsequent ligand screening and binding mode analysis.

In addition, to comprehensively analyze the stability and conformational behavior of protein–ligand complexes, a series of structural parameters was calculated using the GROMACS toolkit. Specifically, gmx rms, gmx rmsf, gmx gyrate, and gmx sasa were employed for analysis [44]. These results collectively elucidate the binding mechanisms and stability characteristics between ligands and the KRAS G12D mutant protein, providing solid theoretical support for further structural optimization and activity prediction.

#### 3.3.4. Principal Component Analysis

Principal component analysis (PCA) was performed to investigate differences in the protein’s conformational dynamics across ligand-bound systems. All analyses were carried out using GROMACS 2025.2.

Prior to PCA, molecular dynamics trajectories of all systems were preprocessed. Considering that the total number of atoms varied among different ligand systems, only the Cα atoms of the protein were extracted to ensure comparability. Specifically, the gmx trjconv module was employed to select Cα atoms from the original trajectories, generating reduced trajectory files for subsequent analysis.

Subsequently, the Cα trajectories from all systems were processed in a unified manner. To construct a common conformational space, the trajectories were concatenated sequentially using the gmx trjcat tool. During concatenation, the continuous-time mode (-settime) was used to maintain temporal continuity across trajectory segments.

After generating the combined trajectory, the initial structure of the reference system was selected as the alignment structure, and its Cα coordinates were extracted as the reference. Based on this reference, the covariance matrix of atomic positional fluctuations was calculated from the merged trajectory, and the resulting matrix was decomposed using the gmx covar module to obtain eigenvalues and eigenvectors.

The combined trajectory was then projected onto the first two principal components (PC1 and PC2) using the gmx anaeig tool, yielding the distribution of each frame in the principal component space. To ensure numerical stability, the number of OpenMP threads was set to one during the analysis.

Finally, scatter plots based on PC1 and PC2 projections were generated to visualize and compare the conformational distributions of the two mechanistic systems.

## 4. Conclusions

In this study, we developed a deep learning framework based on protein–compound feature fusion (MPFF-IS) for the identification of potential inhibitors targeting the KRAS G12D mutant. By integrating ESM2-based protein sequence representations with MPNN-derived molecular graph features, the model effectively captures protein–ligand interaction information and demonstrates strong predictive performance on the KRAS G12D dataset.

Using this model, we performed large-scale virtual screening of 133,856 compounds and identified 2663 high-confidence candidate molecules. Representative ligands were further selected based on molecular docking results, and their structural stability and dynamic behavior were systematically evaluated through 300 ns molecular dynamics simulations. The results indicate that several candidate compounds exhibit comparable or improved performance relative to reference inhibitors (such as MRTX1133 and BI-2852) in key metrics, including RMSD, RMSF, and binding free energy. Meanwhile, these compounds form stable hydrogen-bond networks within the Switch I/II regions and the GDP-binding pocket and display enhanced hydrophobic and π–π/π–cation interactions. This enables effective recognition of key residues and cooperative interaction patterns in binding. Collectively, these findings support the potential binding capability and stability of the identified candidates from both structural and dynamic perspectives.

It should be noted that this study has several limitations. First, the current model is designed to predict inhibitory activity against a single protein target. In recent years, inhibitors that engage multi-protein cooperative mechanisms (e.g., RMC-6236, which binds to CypA and subsequently interacts with KRAS G12D, thereby showing improved inhibitory activity [45]) have emerged as an important direction in drug development. Due to fundamental differences between multi-target inhibition and single-target binding in terms of molecular recognition and functional regulation [46], the current model architecture and training data do not capture such complex scenarios. Addressing this limitation will require developing new models that integrate protein complex structures and network pharmacology information, representing an independent direction for future research.

Overall, this study provides a feasible and extensible computational framework for the efficient screening of KRAS single-target inhibitors, laying a foundation for subsequent drug design efforts in this area.

## Figures and Tables

**Table 1:** Model performance on the KRAS G12D dataset.

KRAS G12D Dataset	Accuracy	ROC-AUC	F1	MCC	Recall	Precision	Specificity
MPFF-IS	0.8465	0.9463	0.8040	0.6881	0.8889	0.7339	0.8232
KNN [26]	0.6496	0.7363	0.6454	0.3963	0.8804	0.5094	0.5185
SVM [26]	0.7047	0.8176	0.5856	0.3565	0.5761	0.5955	0.7778
RF [26]	0.7244	0.8235	0.6500	0.4288	0.7065	0.6019	0.7346
GNB [27]	0.5743	0.6095	0.5574	0.2163	0.7391	0.4474	0.4815
XGB [28]	0.7559	0.8356	0.6837	0.4886	0.7283	0.6442	0.7716
LightGBM [28]	0.7835	0.8395	0.7120	0.5398	0.7391	0.6869	0.8086
CatBoost [28]	0.7441	0.8411	0.6766	0.4720	0.7391	0.6239	0.7469
DeepDTA [29]	0.7637	0.8246	0.6739	0.4887	0.6739	0.6739	0.8148
GraphDTA [30]	0.7401	0.7842	0.6250	0.4278	0.5978	0.6547	0.8209

**Table 2:** MMPBSA calculation results for GDP-state stabilizing inhibitors.

Lig	ΔG_bind_	ΔEEL	ΔVDWAALS	ΔEPB	ΔENPOLAR
MRTX1133	−54.91	−40.06	−69.60	60.18	−5.43
Lig1	−41.92	−33.84	−56.84	53.63	−4.86
Lig2	−53.61	−34.01	−67.11	52.71	−5.18
Lig3	−54.34	−34.81	−71.31	56.10	−5.33

**Table 3:** MMPBSA calculation results for GDP/GTP-binding type inhibitors.

Lig	ΔG_bind_	ΔEEL	ΔVDWAALS	ΔEPB	ΔENPOLAR
BI2852	−25.07	−40.67	−35.09	54.30	−3.60
Lig4	−29.83	−17.48	−49.71	42.02	−4.66
Lig5	−30.00	−20.01	−48.93	43.81	−4.86
Lig6	−16.27	−15.29	−26.08	27.66	−2.56

## Data Availability

The original contributions presented in this study are included in the article/Supplementary Material. Further inquiries can be directed to the corresponding author.

## Supplementary Materials

The following supporting information can be downloaded at: https://www.mdpi.com/article/10.3390/ph19050753/s1.
