# Supplementary material for: A Molecular–Protein Fusion Framework for Rapid Virtual Screening: Accelerating Lead Discovery for “Undruggable’’ Oncogenic Targets
Source: Pharmaceuticals (Basel). 2026 May 12;19(5):753. doi: 10.3390/ph19050753 (PMC13209736; doi:10.3390/ph19050753)
Supplement: Supplementary file 1 [file pharmaceuticals-19-00753-s001.zip › pharmaceuticals-4274069-supplementary.pdf]

## Supplementary Materials

All models, code, and datasets are available at the following website:
https://github.com/Frozen-Zephyr/MPFF-IS
